# Seropositive Wild Boars Suggesting the Occurrence of a Wild Cycle of *Trichinella* spp. in Brazil

**DOI:** 10.3390/ani12040462

**Published:** 2022-02-13

**Authors:** Carolina S. Silva, Talita O. Mendonça, Dália M. R. Machado, Carmen A. Arias-Pacheco, Wilson J. Oliveira, Patricia P. Perin, Karin Werther, Paulo E. Carraro, Iara M. Trevisol, Beatris Kramer, Virgínia S. Silva, Luis A. Mathias, Karina P. Bürger, Estevam G. Lux Hoppe

**Affiliations:** 1 Parasitic Diseases Laboratory (LabEPAr), Departament of Pathology, Reproduction, and One Health (DPRSU), São Paulo State University—UNESP, Agrarian and Veterinarian Sciences (FCAV), Jaboticabal 14884-900, SP, Brazil; carolina.santos-silva@unesp.br (C.S.S.); talita.mendonca@unesp.br (T.O.M.); dalia.machado@unesp.br (D.M.R.M.); carmen.arias@unesp.br (C.A.A.-P.); junior.oliveira@unesp.br (W.J.O.); patricia.perin@unesp.br (P.P.P.); karin.werther@unesp.br (K.W.); ducarrarovet@hotmail.com (P.E.C.); la.mathias@unesp.br (L.A.M.); karina.burger@unesp.br (K.P.B.); 2 Animal Genetics and Health Laboratory (LSGA), Embrapa Swine and Poultry, BR-153 Road, Km 110, Tamanduá District, Concórdia 89715-899, SC, Brazil; iara.trevisol@embrapa.br (I.M.T.); beatris.kramer@embrapa.br (B.K.); virginia.santiago@embrapa.br (V.S.S.)

**Keywords:** *Trichinella* spp., wild boars, wild carnivores, iELISA, artificial digestion

## Abstract

**Simple Summary:**

Trichinellosis is an important zoonotic disease with worldwide distribution and two types of cycles: the domestic cycle and the wild cycle, and spillover between them can occur occasionally. There are several cases of human and animal infection by *Trichinella* spp. in South America, but Brazil is considered free of the domestic cycle, even though there is unpublished serological evidence of *Trichinella* spp. in wild boars. This study investigates *Trichinella* spp. infection in wild boars and wild carnivores in Brazil by serological and artificial digestion tests. We tested 136 samples: 121 from wild boars and 15 from wild carnivores, the latter only by artificial digestion. No larvae were found in the artificial digestion tests, but 6.1% (95% CI 3.0–12.0) of the wild boars were positive in iELISA. These results suggest the occurrence of a *Trichinella* spp. wild cycle related to wild boars in Brazil, but further analyses should be performed to confirm the presence of the parasite.

**Abstract:**

*Trichinella* is a zoonotic nematode traditionally detected worldwide in both domestic and wild animals. In South America, along with the occurrence of this parasite in domestic pigs and wild boars, there are reports of infection in wild carnivores. Brazil is considered free of the domestic cycle of *Trichinella*, but there is unpublished serological evidence of infection in wild boars, which changed the Brazilian status in OIE regarding the disease after an official communication. We investigated *Trichinella* spp. infection in wild boars and wild carnivores in the Southeastern region of Brazil. A total of 136 samples were tested, 121 from wild boars and 15 from wild carnivores. Artificial enzymatic digestion (AED) tests were performed on muscle samples from 37 wild boars and 15 wild carnivores, and 115 serum samples from wild boars were tested by iELISA. Seven serum samples from wild boars tested positive (7/115 = 6.1%, 95% CI 3.0–12.0), but no larvae were found in the AED. There was no significant difference between sex, age, and location of the samples. The serological results suggest that a wild cycle of *Trichinella* spp. may occur in Brazil, but further analyses should be performed to confirm the presence of the parasite.

## 1. Introduction

Trichinellosis is caused by nematodes of the genus *Trichinella*, one of the most widespread zoonotic pathogens in the world. These parasites have been reported in domestic and wild animals on all continents except Antarctica [1]. Currently, ten *Trichinella* species and three genotypes are recognized, and they are divided in two clades: encapsulated and non-encapsulated [2]. The former comprises seven species, with their respective genotypes in parentheses: *Trichinella spiralis* (T1), *Trichinella nativa* (T2), *Trichinella britovi* (T3), *Trichinella murrelli* (T5), *Trichinella nelsoni* (T7), *Trichinella patagoniensis* (T12), and *Trichinella chanchalensis*, along with three genotypes, namely T6, T8, and T9. All of the encapsuled species are known to affect mammals and lead to the formation of modified host cells called “nurse cells”. The non-encapsuled clade is represented by *Trichinella pseudospiralis* (T4), *Trichinella papuae* (T10), and *Trichinella zimbabwensis* (T11). These species are mainly related to reptiles and birds and lost the capability to form nurse cells [3]

Carnivorism is essential for the *Triquinella* spp. life cycle as hosts acquire the parasite by ingesting muscle tissue containing infective larvae [2,3,4]. There are two different epidemiological cycles: the domestic cycle, related to urban and rural settings and consisting of transmission between domestic and synanthropic animals, in which rodents and pigs are the main hosts and *Trichinella spiralis* the most frequently associated species; and the sylvatic cycle, which involves mainly carnivores and wild boars as the most frequent hosts and includes several species of *Trichinella* of both clades. The transmission patterns in this type of cycle are quite diverse, as they are influenced by geographic region, temperature, altitude, host and parasite species involved, and human behavior [5,6,7].

Wild mammals act as the main reservoirs of *Trichinella* spp. given the higher biomass of the parasite in this group of animals [8], but domestic animals are the most related to human cases [2,9,10]. Spillover between domestic and wild cycles can occur, often being related to human activities [11]. The occurrence of trichinellosis in humans is strongly related to cultural practices that involve eating raw or undercooked meat. Even though it has been historically associated with pork consumption, in many countries human infection results from ingestion of infected meat from other animals, including game [7,12,13,14,15,16,17].

Brazil is one of the South American countries most affected by wild boars, an invasion that has been recorded in recent decades [18,19]. Wild boars are one of the most relevant invasive species due to their deleterious impacts on the environment [20]. Hunting is the main strategy to control the wild boar population in Brazil [18], and as a result, the consumption of wild boar meat has become commonplace among hunters and their social circle, despite the fact that the commercialization of wild boar meat is prohibited. Due to the lack of sanitary control, there is a considerable risk of foodborne diseases.

In South America, four *Trichinella* species have been diagnosed: *T. spiralis* in domestic pigs from Argentina and wild boars and cougars from Argentina and Chile; *T. pseudospirallis* in domestic swine from Argentina; *T. patagoniensis* in Argentinian cougars; and T. britovi in sausages related to human outbreaks [2]. Brazil is considered free of the domestic cycle of *Trichinella* spp., but an unpublished seroepidemiological study revealed seropositive wild boars in indirect ELISA test, changing the country’s OIE disease status in wild animals from “never reported” to “infection in limited zones” [19]. The present study aims to investigate the infection by *Trichinella* spp. in carnivores and wild boars, considering their role in the wild cycle of these parasites, and to determine the occurrence of a wild cycle of *Trichinella* spp. in the studied area.

## 2. Materials and Methods

The study was conducted in São Paulo State and covered an area of approximately 43,000 km^2^. The estimated population of the area is 4,000,000 people, according to the 2010 IBGE Census [21]. In this region, there is a predominance of sugarcane, citrus, peanut, and corn crops. The vegetation cover is represented by Atlantic rainforest and Cerrado savanna [22]. The climate in the studied region is humid tropical (Aw, in the Köppen classification), characterized by significantly warmer temperatures and quite dry winters [23].

The collection of wild boar samples was accomplished from 2018 to 2020 in 13 cities from São Paulo State. Initially, only blood samples were collected, as the carcasses could not be necropsied due to logistical problems related to the hunters’ teams, which hindered the necroscopic examination. Later, these issues were solved and fragments of tongue, masseter, and diaphragm were collected along with the blood samples. The field activities were suspended in March 2020 due to the COVID-19 pandemic.

The wild boars were separated by sex and age based on tooth eruption pattern [24,25]. The animals were classified as younglings when aged less than 12 months old, adults when between 12 and 36 months old, and old adults when aged more than 36 months old. In total, we obtained samples from 121 wild boars, 84 from which only blood samples could be obtained, 31 from which blood and muscle tissue samples could be obtained, and six from which only tissue samples were obtained due to advanced hemolysis.

The sampling of wild carnivores occurred from 2018 to March 2020. We collected fragments of tissues from 15 road-killed animals found in Matão, Ribeirão Preto, Ibitinga, Jaboticabal, Santa Ernestina, Igarapava, Ituverava, Bebedouro, Araraquara, Sertãozinho, and Catanduva. The muscle samples were stored at 2–8 °C for up to five days until analysis. Blood samples could not be obtained due to the carcasses’ conditions.

The blood samples were centrifuged for serum extraction, and the obtained serum samples were stored at −20 °C until analysis. The serum samples were analyzed by indirect ELISA test validated for wild boars (ID Screen^®^ *Trichinella* Indirect Multi-species Kit, IDvet, Grabels, France) with high sensibility and specificity. As *Trichinella* infection is a notifiable disease, the tests were developed at the Laboratory of Animal Genetics and Health (LSGA) of the Embrapa Swine and Poultry, in Concórdia, Santa Catarina State. This is the only Brazilian laboratory authorized and recognized by the Ministry of Agriculture, Livestock and Supply (MAPA) for the serological diagnosis of *Trichinella* infection in wild boars in Brazil. The samples were processed following the manufacturer’s instructions and using control serum provided in the commercial kit.

For wild boars, we used 20 g of diaphragm, tongue, and/or masseter muscles. For the wild carnivores, 20 g of the forearm muscle, tongue and/or diaphragm was used. The artificial enzymatic digestion technique was performed according to Gamble et al. (2000) [26].

The 95% confidence intervals (95% CIs) of the obtained prevalence rates were calculated using the Wilson method. Fisher´s exact test was used to verify the association between the prevalence rates and the variables of host sex, host age, and host locality. The confidence intervals and Fisher’s exact test were determined using the R “binom” package, and the level of significance was 0.05.

## 3. Results

The total sampling effort of the team during the study period was of 960 h, resulting in 0.12 animals/hour. In addition, we obtained 15 carcasses of road-killed carnivores in roads from the region. Data on the obtained specimens, samples, and tests are detailed in Table 1.

**Figure 1:**
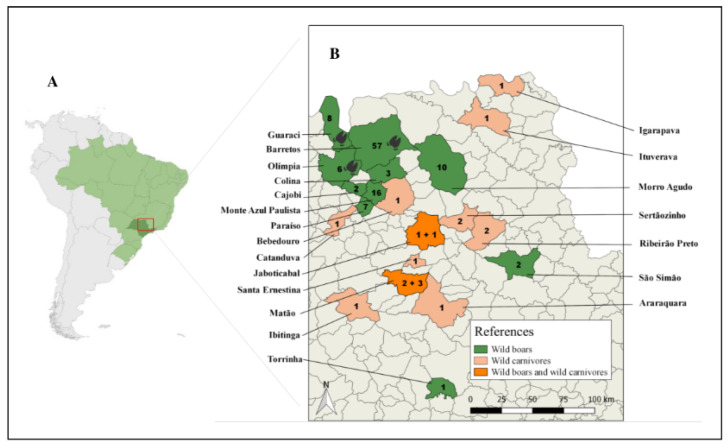
Map of South America (**A**). Location of the study area, distribution of animal groups, and sampled cities in southeastern Brazil (**B**). The numbers indicate the number of animals examined in each city: only wild boars, wild boars and wild carnivores, and only wild carnivores. The symbol 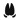 represents cities with seropositive wild boars.

The muscle tissues from 37 wild boars and 15 carnivores were submitted to the artificial enzymatic digestion test in search of *Trichinella* spp. larvae, but all tested negative. From the 115 serum samples of wild boars tested, seven were positive for *Trichinella* spp. (7/115 = 6.1%, 95% CI (3.0–12.0)): four females (04/56 = 7.1%, 95% CI (2.8–17.0)) and three males, (03/59 = 5.1%, 95% CI (1.7–13.9)), in the cities of Barretos (05/57 = 8.8%, 95% CI (0.0–18.9)), Guaraci (01/08 = 12.5%, 95% CI (0.0–47.1)), and Olímpia (01/06 =16.7%, 95% CI (3.0–56.3)) (Table 2, Figure 1). According to the statistical analyses, there were no significant differences between the prevalence of *Trichinella* spp. in the different cities, in the different age groups, or between males and females (Table 2 and Table 3).

## 4. Discussion

Trichinellosis has already been described in Argentina and Chile, and five countries in South America have reported the infection in animals by direct or indirect methods [27]. The observed seroprevalence of *Trichinella* spp. in the studied wild boars was 6.1% (7/115, CI95% (3.0–12.0)), without significant association between infection and host age, sex, or locality. Historically, Brazil is free from *Trichinella* spp. infection in synanthropic rodents [28], domestic swine [19,29,30,31,32], and horses [33,34], and the disease was never reported in humans. However, even though the OIE recognizes Brazil as free from the domestic cycle of *Trichinella* spp., serosurveillance in wild boars from São Paulo, Mato Grosso, Santa Catarina, and Rio Grande do Sul States revealed seropositive animals in the iELISA test, changing the country’s status for the wild cycle, after an official communication by the government agencies [19].

The global seroprevalence of *Trichinella* spp. in wild boars, the second most important source of infection of this disease to humans, is estimated at 6% [35]. The present study showed similar prevalence, 6,1% (95% CI 3.0–12.0), and the absence of significant association between *Trichinella* spp. infection and host age, sex, or site of collection. Although the applied statistical tests showed the absence of association between the variables, in some cities a smaller number of samples was collected when compared to others. The three cities where the samples of the seropositive animals were obtained are geographically close, and perhaps a larger and more uniform sampling would show a different result.

A meta-analysis of *Trichinella* infection in North America, Europe, Asia, and Oceania indicated an absence of interactions between these factors, but in Argentina, even though there were no differences between the age and sex of the host, the prevalence of infection in wild boars from different Patagonia regions was probably influenced by anthropic action due to the presence of cultivated areas [36]. In the present study, all sampled wild boars were captured in strongly anthropized areas, mainly close to large sugarcane and citrus plantations, both inside the cultivated areas or in the surrounding forest fragments.

The indirect ELISA test has high sensitivity and specificity; it is the recommended serological method for screening large numbers of animals for surveillance by the World Organization for Animal Health [37]. The commercial kits are suitable for detection of anti-*Trichinella* spp. Antibodies, both in serum and meat juice samples, representing a reliable tool for serosurveillance of this parasite in domestic species. When compared to direct methods such as enzymatic digestion, the ELISA test exhibits higher sensitivity in samples with low parasite loads but has reduced ability to detect antibodies in recently infected animals, even when infective larvae are found in the muscle [26,38,39]. We were not able to collect tissue and serum samples of all animals included in this study, and unfortunately we lack data of artificial enzymatic digestion for the seropositive wild boars, preventing the comparison between direct and indirect tests. The Western blot test could be useful in order to discard cross-reaction to parasite species other than *Trichinella* spp., but our laboratory lacks the infrastructure for this test. However, infection by other species of parasites is frequent in these animals, as the studied animals were parasitized by at least one helminth species [unpublished data]. The Elisa test used offers high sensitivity and specificity based on the use of the excretory/secretory (E/S) antigen that allows the detection of antibodies directed against *Trichinella* spp. [manufacturer’s manual] [40], so if nonspecific reactions were related to the positive tests, the observed prevalence could be higher, but our team will continue its surveillance of wild boars in search of *Trichinella* spp. larvae nonetheless.

Regarding the carnivores, ELISA could be an important tool in epidemiological studies on these and other wild species [41]. The validation of serological assays for non-domestic species, however, is hindered by the lack of reference sera, as well as the fact that the samples are more prone to contamination and/or hemolysis, which may cause predisposition to a higher number of false-positive results [42]. Therefore, enzymatic digestion is the most reliable test for surveillance of these parasites in wild carnivores. This diagnostic tool allows for the detection of parasitized animals with one to three larvae per gram of tissue [43,44,45]. This method was successfully used to identify *Trichinella* spp. in autochthonous and exotic wild animals from Argentina [46,47] and Chile [48,49,50], even allowing the discovering of a novel species, *Trichinella patagoniensis*, in Argentinean cougars [51].

The negative results observed on enzymatic digestion, both for wild carnivores and wild boars, may be related to a low larval load and/or low prevalence—or even absence—of the parasite in the samples evaluated. When we consider the expected prevalence for the enzymatic digestion technique, based on the prevalence obtained in the serology technique, it was expected that the enzymatic digestion technique could detect at least two positive samples (6.1% of 37 samples analyzed). There is still the possibility that the population is infected with a species that produces a very low parasite load, such as what happens with *T. patagoniensis*, which produces a low parasite load in domestic swine and wild boar hosts [37,51,52,53]. The analysis of samples from wild carnivores by artificial enzymatic digestion was the first study performed in Brazil. The negative results are in line with negative results reported for other wild species such as white-eared opossums (*Didelphis albiventris*), hairy armadillos (*Chaetophractus vellosus*), Geoffroy’s cats (*Leopardus geoffroyi*), ferrets (*Galictis cuja*), Molina’s hog-nosed skunk (*Conepatus chinga*), and Pampas foxes (*Lycalopex gymnocercus*) from the northeastern region of Argentinean Patagonia [54].

As for human infection, Brazilians traditionally do not have the cultural habit of eating raw, undercooked, or smoked pork or game meat, and end up being less exposed to the risk of transmission. Argentines and Uruguayans have very strong traditions on the preparation and consumption of raw and smoked pork or wild boar meat products (raw ham, chorizo, bacon), which increases the risk of exposure to *Trichinella* [55]. A survey with registered hunters from São Paulo State, however, revealed that 84.2% of them consume wild boar meat, and even though they recognize that game meat can represent a health risk, 15.8% prefer undercooked meat. In addition, 26.3% of the hunters reported using game meat for the manufacture of products such as sausages and salami, thus presenting a potential risk to human and animal health [56]. The cultural habit of consuming hunted wild boar meat in Brazil is recent, since hunting this species has only been allowed in 2013 [57] and may represent a change in human behavior and the introduction of new eating habits, which could lead to increased exposure to and risk of contracting trichinellosis.

Regarding animal health, wild boars can be a potential source of *Trichinella* spp. for production systems located within the radius of occupancy of their populations [58]. Although commercial pig farming in Brazil follows strict internal biosecurity standards [59], it is known that there are more than 800 home-based or subsistence-type farms with low confinement, as well as free-range/organic animal production in São Paulo state [60,61], similar to what occurs in Argentina [62,63]. These production systems are more prone to spillover from the wild cycle of *Trichinella*.

Brazil still does not have a structured and well-defined surveillance program for *Trichinella* spp. in wild boars or other wild animals. There is a partnership between Embrapa Swine and Poultry and the Ministry of Agriculture, Livestock and Supply (MAPA), with a proposal for structuring an epidemiological surveillance system as a complement to the Health Surveillance System for Classical Swine Fever (CSF) [64]. With this partnership, Embrapa currently performs serological diagnostics for *Trichinella* spp. in all serum samples that are sent by the Regional Units of Agricultural Defense and go through the CSF program, and the results are reported to MAPA, which is responsible for notification to the World Organization for Animal Health (OIE). Registered hunters, who voluntarily collect and forward the biological material, provide these samples to the regional units, making them important agents for the surveillance of wild boars.

## 5. Conclusions

The wide distribution of wild boars and the presence of seropositive animals for *Trichinella* spp. suggest that a wild cycle may occur in Brazil, which, with new patterns of human behavior, increasing changes in eating habits, and changing agricultural practices, may be a determining factor in the increased risk of exposure in domestic animals and humans to this parasite. Even though, further studies are needed to search for the parasite larvae in order to effectively confirm the occurrence of *Trichinella* spp. in Brazil and determine the circulating species.

## Figures and Tables

**Table 1:** Animal species and number of samples evaluated by iELISA, iELISA, and enzymatic digestion (iELISA + ED), and enzymatic digestion (AED).

Species	N	Host Characteristics			iElisa	iElisa + AED	AED	TOTAL
		M	F	Age				
Wild boars (*Sus scrofa*)	121	61	60	46YL/69A/06OA	84	31	06	121
Cougars (*Puma concolor*)	05	04	01	05 A	-	-	05	05
Ocelots (*Leopardus pardalis*)	03	03	00	03 A	-	-	03	03
Maned wolves (*Chrysocyon brachyurus*)	02	01	01	02 A	-	-	02	02
Crab-eating foxes (*Cerdocyon thous)*	04	01	03	04 A	-	-	04	04
Southern little spotted cat (*Leopardus guttulus*)	01	01	00	01 A	-	-	01	01
**TOTAL**					84	31	21	136

N—number of animals; M—Males; F—Females; YL—Younglings; A—Adults; OA—Old adults.

**Table 2:** Seroprevalence of *Trichinella* spp. antibodies in wild boars according to location.

Location	N	Prevalence (%)	IC 95% (%)
Barretos	57	8.8 (5/57)	0.0–18.9
Cajobi	2	0	0.0–65.8
Colina	3	0	0.0–56.1
Guaraci	8	12.5 (1/8)	0.0–47.1
Jaboticabal	1	0	0.0–79.3
Matão	2	0	0.0–65.8
Monte Azul	16	0	0.0–19.3
Morro Agudo	10	0	0.0–27.7
Olimpia	6	16.7% (1/6)	3.0–56.3
Paraíso	7	0	0.0–35.4
São Simão	2	0	0.0–65.8
Torrinha	1	0	0.0–79.3
TOTAL	115	6.1 (7/115)	3.0–12.0

No significant differences between sampling localities were observed in Fisher’s exact test (*p* = 0.74).

**Table 3:** Seroprevalence of *Trichinella* spp. antibodies in wild boars according to age and sex.

Host Characteristics	N	Prevalence (%)	CI 95%
Age			
Younglings	44	11.4 (5/44)	4.9–24.0
Adults	65	3.1 (2/65)	0.8–10.5
Old adults	6	0	0.0–39.0
Sex			
Females	56	7.1 (4/56)	2.8–17.0
Males	59	5.1 (3/59)	1.7–13.9

No significant differences were observed in Fisher’s exact test between prevalence and host age (*p* = 0.23) or sex (*p* = 0.71).

## Data Availability

Not applicable.
